# Diagnostic values of soluble triggering receptor expressed on myeloid cells (sTREM-1) and interferon-inducible protein-10 (IP-10) for severe mycoplasma pneumoniae pneumonia in children

**DOI:** 10.1016/j.clinsp.2024.100361

**Published:** 2024-04-27

**Authors:** Chang Xu, Li-Yan Luo, Bi-Chen Wu, Niu Ding, Shi-Jie Jin, Jian-Bao Huang, Yan-Ping Chen

**Affiliations:** Department of Respiratory, Hunan Children's Hospital, Changsha, China

**Keywords:** sTREM-1, IP-10, Severe mycoplasma pneumoniae pneumonia, Diagnostic value

## Abstract

**Objective:**

Early diagnosis of Severity Mycoplasma Pneumoniae Pneumonia (SMPP) has been a worldwide concern in clinical practice. Two cytokines, soluble Triggering Receptor Expressed on Myeloid cells (sTREM-1) and Interferon-Inducible Protein-10 (IP-10), were proved to be implicated in bacterial infection diseases. However, the diagnostic value of sTREM-1 and IP-10 in MPP was poorly known. This study aimed to investigate the diagnostic value of sTREM-1 and IP-10 for SMPP.

**Methods:**

In this prospective study, the authors enrolled 44 children with MPP, along with their clinical information. Blood samples were collected, and cytokine levels of sTREM-1 and IP-10 were detected with ELISA assay.

**Results:**

Serum levels of sTREM-1 and IP-10 were positively correlated with the severity of MPP. In addition, sTREM-1 and IP-10 have significant potential in the diagnosis of SMPP with an Area Under Curve (AUC) of 0.8564 (*p*-value = 0.0001, 95% CI 0.7461 to 0.9668) and 0.8086 (*p-value* = 0.0002, 95% CI 0.6918 to 0.9254) respectively. Notably, the combined diagnostic value of sTREM-1 and IP-10 is up to 0.911 in children with SMPP (*p-value* < 0.001, 95% CI 0.830 to 0.993).

**Conclusions:**

Serum cytokine levels of sTREM-1 and IP-10 have a great potential diagnostic value in children with SMPP.

## Highlights

• The serum levels of sTREM-1 and IP-10 were increased in children with mycoplasma.
• Serum levels of sTREM-1 and IP-10 were positively correlated with the severity of the disease.
• sTREM-1 combined with IP-10 has an important value in the diagnosis of children with MPP.

## Introduction

Mycoplasma Pneumoniae (MP) is among the smallest self-replicating bacteria that lack typical bacterial cell walls.^1^ It is a common pathogenetic organism of respiratory infection in children. Mycoplasma Pneumoniae Pneumonia (MPP) accounts for approximately 8% to 40% of Community-Acquired Pneumonia (CAP) in children aged from 3 to 15 with regional epidemics occurring every 3 to 7 years.^2^^,^^3^ Although MPP spreads easily among children who are in close contact with each other, it is typically a self-limited disease. However, Severe MPP (SMPP) happens with serious pulmonary and extrapulmonary complications at times, including pulmonary atelectasis, necrotizing pneumonia, myocardial damage and peripheral embolization, which may result in serious impacts on children's clinical outcome and quality of life. Early diagnosis and prompt treatment are of great significance in reducing the mortality and sequelae of children with SMPP. Therefore, there is an urgent need to identify valid biomarkers that indicate the severity of MPP.

Soluble Triggering Receptors Expressed on Myeloid cell-1 (sTREM-1) is an important inflammatory factor and index of oxidative stress. The increase of its concentration leads to the activation of downstream inflammatory signaling pathways, such as interleukin-6 and interleukin-1β. sTREM-1 cooperates with Toll-Like Receptors (TLRs) and mediates the expansion of inflammatory response, thus promoting the progression of pulmonary infectious disease.^4^, ^5^, ^6^ sTREM-1 shows diagnostic value in diseases such as Neonatal Sepsis.^7^^,^^8^ IP-10, a chemokine that belongs to the CXC family, was induced by interferon in several cell types such as monocytes, neutrophils, fibroblasts and endothelial cells. IP-10 is also a chemoattractant for activated T-cells. IP-10 was detected in many Th1-type inflammatory diseases, where it is considered to play an important role in recruiting activated T cells into sites of tissue inflammation.^9^^,^^10^ It was reported that IP-10 was involved in infection diseases including pulmonary tuberculosis^11^ and lymphoma-associated hemophagocytic syndrome.^12^ IP-10 was also implicated in SMPP, however, the diagnostic value of sTREM-1 and IP-10 in MPP remains to be depicted.

In this prospective study, the authors aimed to explore the relationship between the serum levels of sTREM-1/IP-10 and the severity MPP. The authors also analyzed the diagnosis value of these two cytokines to see if they could act as novel biomarkers for children with SMPP.

## Methods

### Study population

From January to November of 2021, 44 children with MPP from the Hunan Children's Hospital were recruited for this prospective analysis. Criteria for selecting the subjects were as follows: (i) Clinical presentation (fever, cough, tachypnea, abnormal breath sounds) and radiologic evidence of CAP (interstitial infiltrates, segmental and lobar consolidations, hilar lymph node enlargement); (ii) Microbiological evidence from serologic testing, positive Polymerase Chain Reaction (PCR) tests of nasopharyngeal secretions or Bronchoalveolar Lavage Fuid (BALF), indications for bronchoscopy were persistent radiological abnormalities (atelectasis and consolidation of lung felds). Exclusion criteria were the following: (i) Patients with primary or secondary immune deficiency/dysfunction, including congenital heart disease, chronic liver or kidney disease, oncologic disorders, connective tissue disease, chronic lung disease; (ii) Patients in convalescent-phase; (iii) Patients with mixed infection; (iv) Patients diagnosed with severe MPP later during the hospitalization as the disease progressed. The chart of patient selection was displayed.

Patients were categorized into mild MPP group (*n* = 22) and SMPP group (*n =* 22), according to the clinical parameters and laboratory tests on admission.^13^, ^14^, ^15^ Severity MPP was defined based on the criteria of community-acquired pneumonia. The mild MPP group was defined as respiratory rate < 70 breaths/min at age < 3 years old or respiratory rate < 50 breaths/min at age ≥ 3 years old, no dehydration, and normal food intake. Meanwhile, the SMPP group was defined as tachypnea with a respiratory rate ≥ 70 breaths/min at age < 3 years old or respiratory rate ≥ 50 breaths/min at age ≥ 3 years old (without interference from fever and cry), increased work of breathing (faring of the nares, marked retractions, grunting), capillary ref time ≥ 2s, cyanosis, anorexia and dehydration, the appearance of pulmonary and extrapulmonary combinations including pleural effusion, lung necrosis/lung abscess, myocardial damage and peripheral embolization. This study received ethical clearance from the Ethics Committee at Hunan Children's Hospital (Ethics Committee study protocol number: HCHLL-2022-99) in Changsha City. All methods were carried out in accordance with relevant guidelines and regulations. Parents or legal guardians of all the participants provided written informed consent. Detection of serum cytokines levels venous blood samples of each patient were collected on admission. Measurements of cytokine IL-10 and chemokines CCL2, CCL8 in serum were quantified by Human Inflammatory Cytokine ELISA Kit and Human Chemokine Kit (Becton, Dickinson and Company), according to the manufacturer's standard protocol (data not shown). This study conforms to the STARD guidelines.

### Statistical analysis

Statistical analyses were performed with the SPSS software, version 20.0. Continuous variables were summarized as median (interquartile range) while categorical variables were described as proportion. Clinical characteristics that were significant (*p* < 0.1, univariate analysis) were included in the multivariate forward stepwise logistic regression analysis to identify independent influence factors. Diagnostic accuracy was estimated by the Receiver Operating Characteristic (ROC) curve analysis. Spearman rank correlations were used to assess correlations between variables. A *p-value* < 0.05 was considered as statistically significant.

## Results

### Serum levels of sTREM-1 and IP-10 in children with MPP

**Fig. 1:**
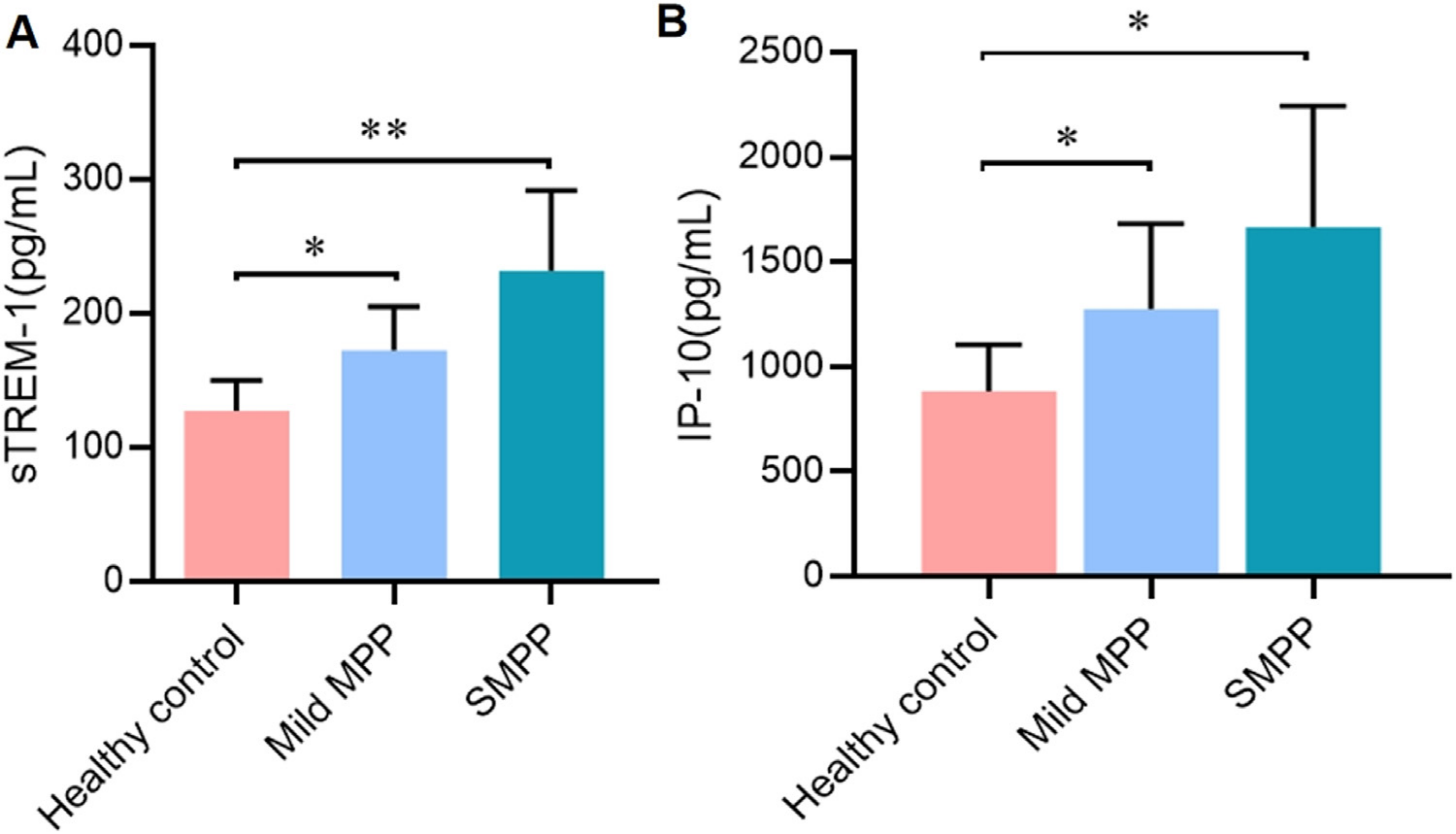
Protein levels of urine soluble triggering receptor expressed on myeloid cells-1 (A) and interferon-inducible protein-10 (B) in serum (*p ≤ 0.05, **p ≤ 0.01).

The serum levels of sTREM-1 and IP-10 in individuals with MPP were detected with enzyme-linked immunosorbent assay. As shown in Fig. 1, both levels of sTREM-1 and IP-10 were significantly increased in subjects of MPP than that in healthy control. Notably, levels of sTREM-1 and IP-10 were positively correlated with the severity of MPP, indicating a role of these cytokines in the pathogenesis of MPP.

### Diagnostic value of serum sTREM-1 and IP-10 levels in children with SMPP

**Fig. 2:**
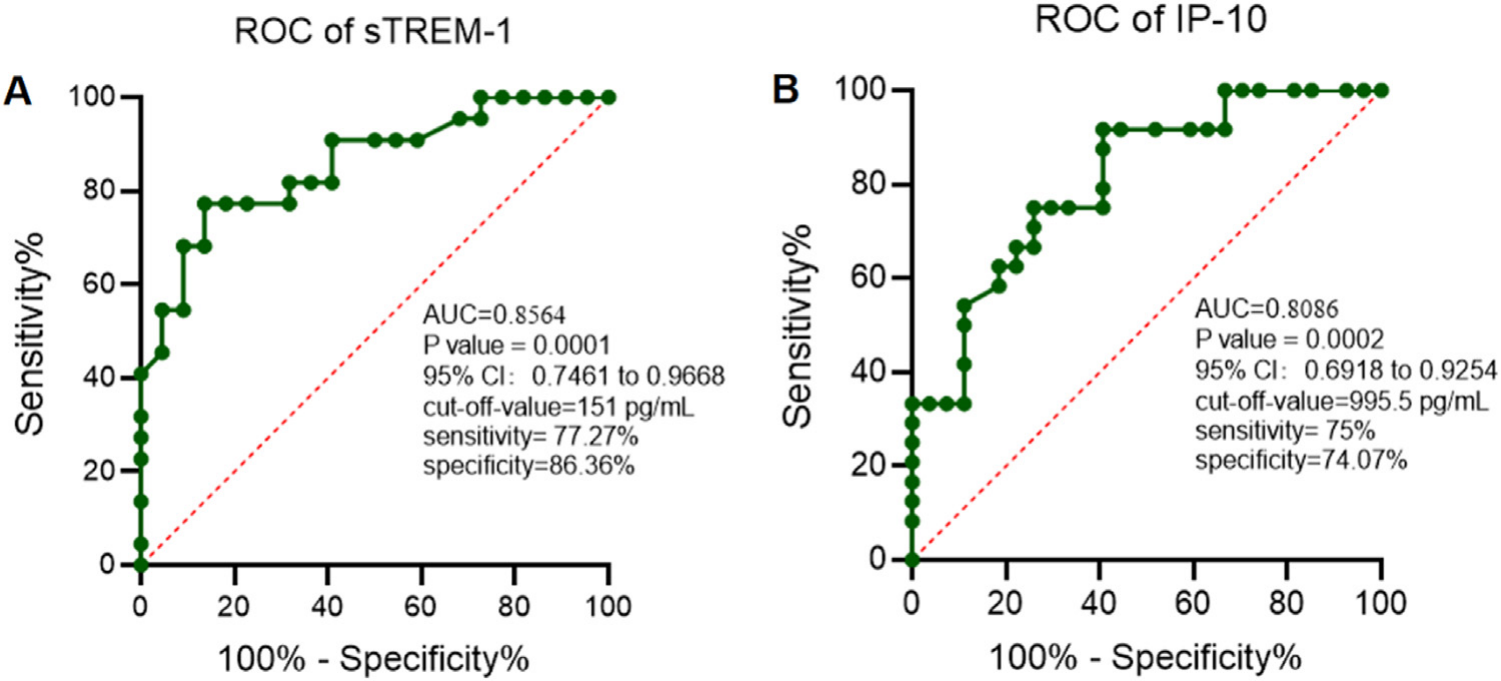
Receiver operating characteristic curve analysis of urine soluble triggering receptor expressed on myeloid cells-1 (A) and interferon-inducible protein-10 (B).

Once sTREM-1 and IP-10 have potential in the diagnosis of infectious diseases, the authors therefore analyzed the diagnosis value of sTREM-1 and IP-10 with the Receiver Operating Characteristic (ROC) curve. As shown in Fig. 2, the Area Under Cure (AUC) for sTREM-1 was 0.8564 with a cut-off-value of 151 pg/mL (*p-value* = 0.0001, 95% CI 0.7461 to 0.9668) and AUC for IP-10 was 0.8086 with a cut-off-value of 995.5 pg/mL (*p-value* = 0.0002, 95% CI 0.6918 to 0.9254). These data suggested that serum levels of sTREM-1 and IP-10 may have significant diagnosis value in children with SMPP.

### Diagnostic value of serum sTREM-1 and IP-10 in children with SMPP

**Fig. 3:**
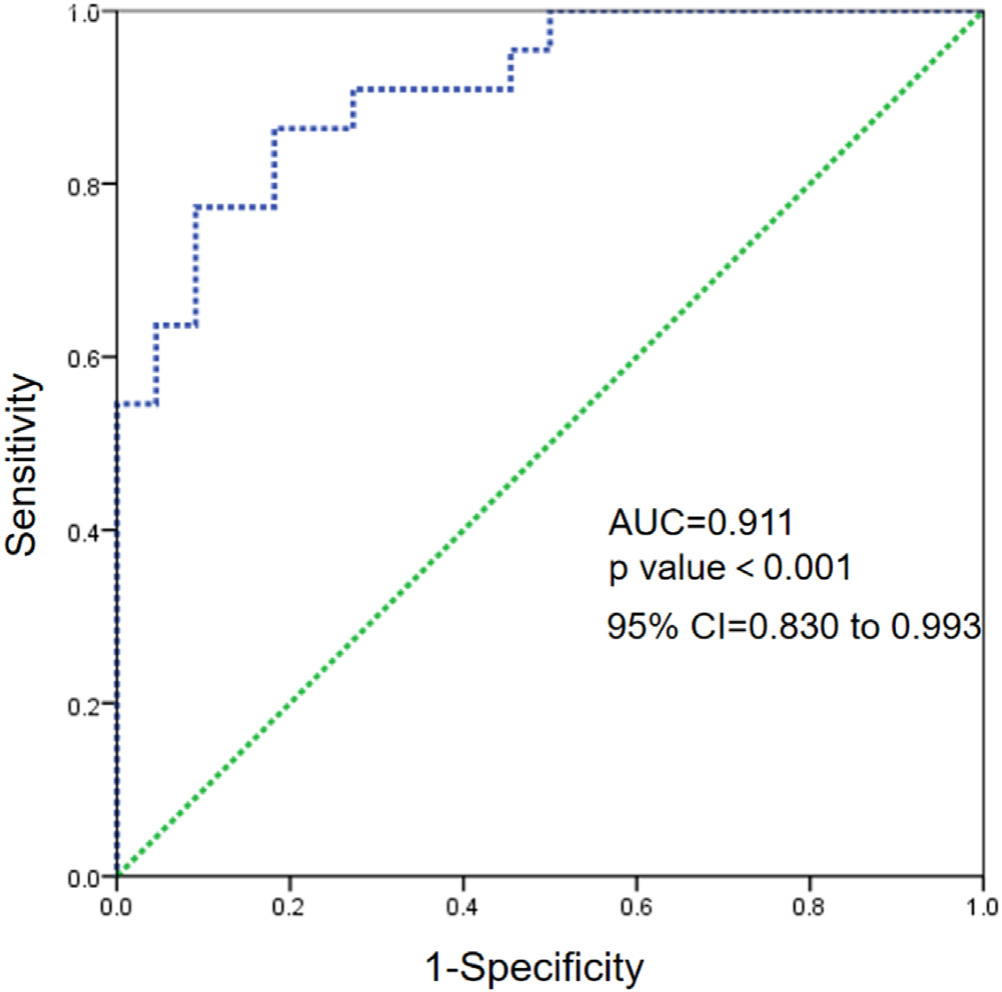
Receiver operating characteristic curve analysis of the combination of urine soluble triggering receptor expressed on myeloid cells-1 and interferon-inducible protein-10.

To make the diagnostic value of serum sTREM-1 and IP-10 higher, the authors also analyzed the combined diagnostic value. As shown in Fig. 3, the AUC is 0.911 (*p-value* < 0.001, 95% CI 0.830 to 0.993), indicating a promising value in the diagnosis of SMPP*.*

## Discussion

MPP is the smallest pathogenic organism capable of living independently on a cell-free culture medium between the size of the bacterium and the virus. It causes respiratory diseases in children, including pharyngitis, bronchitis, and pneumonia. It also causes airway hyperresponsiveness, including asthma, as well as numerous extrapulmonary manifestations.^16^^,^^17^ Mycoplasma pneumoniae enters into the body through respiratory tract or contact infection and then grows between ciliated epithelia, inhibiting ciliated activity and destroying epithelial cells to cause local tissue damage, thus causing MPP.^18^^,^^19^ MPP is a common respiratory tract infectious disease in pediatrics. Most of the cases are mild and have a good prognosis. However, in recent years, the reports of SMPP have gradually increased. Its pathogenesis may involve many aspects, such as children of large lactone class antibiotic resistance, excessive immune inflammatory response, diagnostic delays, and mixed infection.^18^^,^^19^ Clinical symptoms are often characterized by severe symptoms, long course of disease, many internal and external pulmonary complications, and poor therapeutic effect, and sequelae such as atelectasis, bronchiectasis obliterans, bronchiolitis obliterans, and other sequelae can be left, which have a serious impact on the physical and mental health of children.^20^^,^^21^ In recent years, with the emergence of resistant strains, the originally effective antimicrobial effect has been reduced and even invalid. Coupled with the combination of factors such as bacteria and virus infection, SMPP cases are increasing. SMPP is easy to merge pulmonary complications and the clinical treatment is challenging. Patients with SMPP have a high mortality risk and there are a lot of bad effects on children's health. Therefore, early diagnosis and timely treatment of mycoplasma pneumonia are of great significance in clinics.

In the present article, the authors enrolled 44 children with MPP and among them 22 patients are diagnosed with SMPP. The authors detected the serum levels of sTREM-1 and IP-10 and find that serum levels of sTREM-1 and IP-10 were positively correlated with the severity of MPP. In addition, the authors used ROC curve analysis to evaluate the diagnosis values of sTREM-1 and IP-10. As expected, levels of sTREM-1 and IP-10 show a potential value in SMPP with an AUC of 0.8564 and 0.8086 respectively. Notably, the combined diagnosis efficiency was much higher with an AUC of 0.911. These data indicate a potential utility of sTREM-1 and IP-10 in the diagnosis of SMPP.

In summary, the levels of serum sTREM-1 and IP-10 in children with MPP are elevated and are positively correlated with the severity of the disease, suggesting that these two cytokines are involved in the pathogenesis of MPP. Furthermore. The ROC curve shows that sTREM-1 combined with IP-10 has a high diagnosis efficiency for children with SMP. Therefore, sTREM-1 and IP-10 may act as feasible biomarkers for the targeted therapy and rapid and early diagnosis of SMPP.

## Ethics approval and consent to participate

Not applicable.

## Consent for publication

The authors declare that all the authors have agreed to publish the article.

## Availability of data and materials

All the data and materials are available.

## Funding

This work was supported by the funding from Scientific Research Project of Hunan Provincial Health Commission (NO.202106010531).

## CRediT authorship contribution statement

**Chang Xu:** Conceptualization, Resources, Supervision, Writing – original draft. **Li-Yan Luo:** Methodology. **Bi-Chen Wu:** Methodology. **Niu Ding:** Investigation. **Shi-Jie Jin:** Investigation. **Jian-Bao Huang:** Conceptualization. **Yan-Ping Chen:** Data curation, Formal analysis, Resources, Supervision.

## Declaration of competing interest

The authors declare that they have no known competing financial interests or personal relationships that could have appeared to influence the work reported in this paper.

## References

[1] Sauteur PMM, van Rossum AM, Vink C. (2014). Mycoplasma pneumoniae in children: carriage, pathogenesis, and antibiotic resistance. Curr Opin Infect Dis.

[2] Yan C, Sun H, Zhao H. (2016). Latest surveillance data on Mycoplasma pneumoniae infections in children, suggesting a new epidemic occurring in Beijing. J Clin Microbiol.

[3] Chen K, Jia R, Li L, Yang C, Shi Y. (2015). The aetiology of community associated pneumonia in children in Nanjing, China and aetiological patterns associated with age and season. BMC Public Health.

[4] Zhao X, Xu L, Yang Z, Sun B, Wang Y, Li G (2020). Significance of sTREM-1 in early prediction of ventilator-associated pneumonia in neonates: a single-center, prospective, observational study. BMC Infect Dis.

[5] Zhang H-F, Zhang X, Sha Y-X, Zhou H-Q, Pan J-H, Xun X (2020). Value of sTREM-1 in serum and bronchoalveolar lavage fluid, APACHE II score, and SOFA score in evaluating the conditions and prognosis of children with severe pneumonia. Zhongguo Dang Dai Er Ke Za Zhi.

[6] Palazzo SJ, Simpson TA, Simmons JM, Schnapp LM. (2022). Soluble triggering receptor expressed on myeloid cells-1 (sTREM-1) as a diagnostic marker of ventilator-associated pneumonia. Respir Care.

[7] Ozdemir SA, Colak R, Ergon EY, Calkavur S. (2020). Diagnostic value of urine strem-1 and urine c-reactive protein for infants with late onset neonatal sepsis. J Pediatric Infect Dis.

[8] Qin Q, Liang L, Xia Y. (2021). Diagnostic and prognostic predictive values of circulating sTREM-1 in sepsis: a meta-analysis. Infect Genet Evol.

[9] Matsukawa A, Hogaboam C, Lukacs N, Kunkel S. (2000). Chemokines and innate immunity. Rev Immunogenet.

[10] Manes TD, Pober JS, Kluger MS. (2006). Endothelial cell-T lymphocyte interactions: iP-10 stimulates rapid transendothelial migration of human effector but not central memory CD4+ T-cells. Requirements for shear stress and adhesion molecules. Transplantation.

[11] El-emiry F, Attia G, Ahmad A, Sakr B. (2016). Diagnostic value of inducible protein-10 in pulmonary tuberculosis. Egyptian J Chest Dis Tuberculosis.

[12] Maruoka H, Inoue D, Takiuchi Y, Nagano S, Arima H, Tabata S (2014). IP-10/CXCL10 and MIG/CXCL9 as novel markers for the diagnosis of lymphoma-associated hemophagocytic syndrome. Ann Hematol.

[13] Platzker A, Chernick V, Boat T, Wilmott R, Bush A. (2006).

[14] Board CMATE. (2013). Guidelines for management of community acquired pneumonia in children (the revised edition of 2013) (II). Zhonghua Er Ke Za Zhi.

[15] Board E. (2013). Guidelines for management of community acquired pneumonia in children (the revised edition of 2013) (I). Zhonghua Er Ke Za Zhi.

[16] Lee K-Y, Youn Y-S, Lee J-W, Kang J-H. (2010). Mycoplasma pneumoniae pneumonia, bacterial pneumonia and viral pneumonia. J Pediatr.

[17] Izumikawa K, Izumikawa K, Takazono T, Kosai K, Morinaga Y, Nakamura S (2014). Clinical features, risk factors and treatment of fulminant Mycoplasma pneumoniae pneumonia: a review of the Japanese literature. J Infect Chemother.

[18] Tomaino J, Keegan T, Miloh T, Kerkar N, Mercer S, Birge M (2012). Stevens-Johnson syndrome after Mycoplasma pneumonia infection in pediatric post-liver transplant recipient: case report and review of the literature. Pediatr Transplant.

[19] Saraya T, Watanabe T, Tsukahara Y, Ohkuma K, Ishii H, Kimura H (2017). The correlation between chest X-ray scores and the clinical findings in children and adults with mycoplasma pneumoniae pneumonia. Intern Med.

[20] She D. (2021). Focus on the difficulties and challenges in the diagnosis and treatment of Mycoplasma pneumoniae pneumonia. Zhonghua Jie He He Hu Xi Za Zhi.

[21] Al Yazidi LS, Hameed H, Kesson A, Herath A, Pandit C, Britton P (2019). A 6-year-old girl with severe, focal Mycoplasma pneumoniae pneumonia. J Paediatr Child Health.

